# Successful Treatment of War Zone Traumatic Lower Extremity Wound With Exposed Tendons Using an Autologous Homologous Skin Construct

**DOI:** 10.7759/cureus.7952

**Published:** 2020-05-04

**Authors:** Owen N Johnson, Michael Nelson, Ivy Estabrooke, Nikolai Sopko, Edward W Swanson

**Affiliations:** 1 Plastic Surgery, Evans Army Community Hospital, Colorado Springs, USA; 2 Surgery, F. Edward Hébert School of Medicine, Uniformed Services University of the Health Sciences, Bethesda, USA; 3 Surgery Wound Care Clinic, Evans Army Community Hospital, Colorado Springs, USA; 4 Government Affairs, PolarityTE, Inc., Salt Lake City, USA; 5 Research and Development, PolarityTE, Inc., Salt Lake City, USA; 6 Medical Affairs, PolarityTE, Inc., Salt Lake City, USA

**Keywords:** autologous homologous skin construct, ahsc, skin graft, free flap, free tissue transfer, trauma, wound, lower extremity trauma, combat injury, military

## Abstract

Extremity injuries are common in contemporary combat and have become more prevalent as fatality rates have dropped to historic lows. Traumatic extremity wounds, especially those sustained in theater, often present with exposed structures such as tendon, bone, and joint, preventing the use of split-thickness skin grafts (STSG) for coverage. Traditional reconstructive options for these complex wounds include skin substitute with delayed STSG, local flaps, debridement of tendons, pedicled distant flaps (such as cross-leg flap), free tissue transfer, and amputation. STSG, whether on top of skin substitutes or after tendon debridement, can result in contracture and functional limitations in the extremities. Flap reconstructions require prolonged procedures, hospital stays, and periods of immobility. As an alternative to traditional reconstructive options, an autologous homologous skin construct (AHSC) uses a small full-thickness elliptical skin harvest from the patient, which is sent to a biomedical manufacturing facility, processed into AHSC, and can be returned and applied to a wound bed as soon as 48 hours after harvest and used up to 14 days after harvest. We present in this case report the treatment of a 42 cm^2^ complex dorsolateral ankle wound with exposed tendons in an active duty soldier following a rollover motor vehicle accident sustained in theater. After application of AHSC, the soldier’s wound closed in nine weeks with pliable, sensate skin. The patient retained function without contractures limiting ankle motion or adhesions limiting tendon gliding. The successful treatment of this complex war zone injury with AHSC has allowed the soldier to quickly participate in unrestricted physical therapy and is on a trajectory for near-term return to active duty.

## Introduction

Advances in military medicine and the introduction of sophisticated armor for individuals and vehicles have significantly reduced the rate of battlefield case fatalities in the post-9/11 conflicts in Afghanistan and Iraq (9.4%) compared to World War II (19.1%) and Vietnam (15.8%) [1-4]. Most injuries (up to 82%) are to the extremities [5]. In addition to combat injuries, more than 30% of all airlift evacuations for medical care from Afghanistan and Iraq between 2001 and 2013 were due to non-battle injuries (NBI) [6]. More broadly, NBI in and out of theater accounted for 34.1% of all casualties between 2003 and 2014, including 18.8% due to vehicle crashes [7]. Of NBI during Operation Enduring Freedom (OEF) and Operation Iraqi Freedom (OIF), 8% in Afghanistan and 11% in Iraq were due to military vehicle-related accidents [6].

Traumatic extremity wounds, especially those sustained in a combat theater, are often complicated due to contamination and exposure of tendons, ligaments, and bone, which eliminates the feasibility of an early split-thickness skin graft (STSG). More complex reconstructions are typically needed for traumatic extremity wounds, such as free tissue transfer, but can result in outcomes that prevent warfighters from returning to duty. Complication rates for free tissue transfer are also high for the treatment of injuries sustained in combat environments due to an extensive zone of injury and in some cases limited available donor sites [8].

An FDA-registered and commercially available autologous homologous skin construct (AHSC; SkinTE^TM^, PolarityTE MD, Inc., Salt Lake City, UT) has shown promise in the treatment of complex wounds [9-13]. AHSC is derived and manufactured from a full-thickness elliptical skin harvest taken from the patient, and returned to the clinician for application to the wound. After AHSC is grafted to the wound bed, it expands over time to regenerate full-thickness skin from the inside out, producing skin that contains all layers (epidermis, dermis, and hypodermis) and appendages including hair follicles and sweat glands [9-14]. Processing of the patient’s skin into AHSC takes advantage of the endogenous regenerative populations within the skin by the creation of optimized multicellular microaggregates including progenitor cells from the hair follicles that are dispersed throughout the product, which is delivered back to the clinician within a syringe in a paste-like substance. Manufacturing of AHSC utilizes the original tissue harvest in its entirety, including all cell populations and extracellular matrix throughout the layers and appendages of skin, and endogenous progenitor cells, providing the necessary components to be able to regenerate full-thickness skin. The optimized microaggregates are able to survive on diffusion within the wound fluid, which allows AHSC to have a longer timeframe of viability prior to needing a direct blood supply. These properties make it feasible to apply AHSC over exposed avascular structures like tendons and bone commonly seen in traumatic injuries. Here we present the first case of AHSC used to treat a complex trauma wound acquired in a combat environment.

## Case presentation

A 38-year-old Caucasian male with a past medical history significant for right plantar fasciitis suffered multiple lower extremity injuries following a motor vehicle rollover accident (MVA) while an active duty army non-commissioned officer serving in Iraq. Of note, he was an active, long-time cigarette smoker at the time of injury. Injuries to bilateral lower extremities were sustained from the MVA, including of the right lower leg, right foot and ankle, left knee, and left lower leg. Open joints were suspected in the right ankle and left knee. A left torus fibular fracture and a right malleolus fracture were confirmed on a plain radiograph. The patient underwent initial debridement and two-incision, four-compartment fasciotomies for compartment syndrome. Screening for traumatic brain injury was negative. Following stabilization and evacuation, first to Germany and then to the United States, the acute injuries were treated with serial debridements, delayed primary closures, and open reduction and internal fixation of fractures with plates and screws. Eventually the last remaining untreated injury was a 7 x 6 cm (42 cm^2^) right dorsolateral ankle wound with multiple exposed extensor tendons.

Intermediate treatment of the wound included serial debridements and local wound care followed by the application of bilayer collagen-GAG matrix (Integra® Meshed Dermal Regeneration Template, Integra LifeSciences, Plainsboro, NJ) one week post-injury. The patient was pessimistic about all traditional treatments options, such as STSG, cross-leg flap, and free-tissue transfer for both donor site morbidity and functional outcome reasons. His goal was to regain normal ankle, foot, and toe function with minimal downtime and a quick return to active duty. Following a comprehensive discussion of treatment options, he chose to pursue treatment with AHSC for coverage of the remaining exposed tendons and epithelial closure due to the minimal donor site impact (5 cm linear groin incision) and the potential for functionally normal skin.

**Figure 1:**
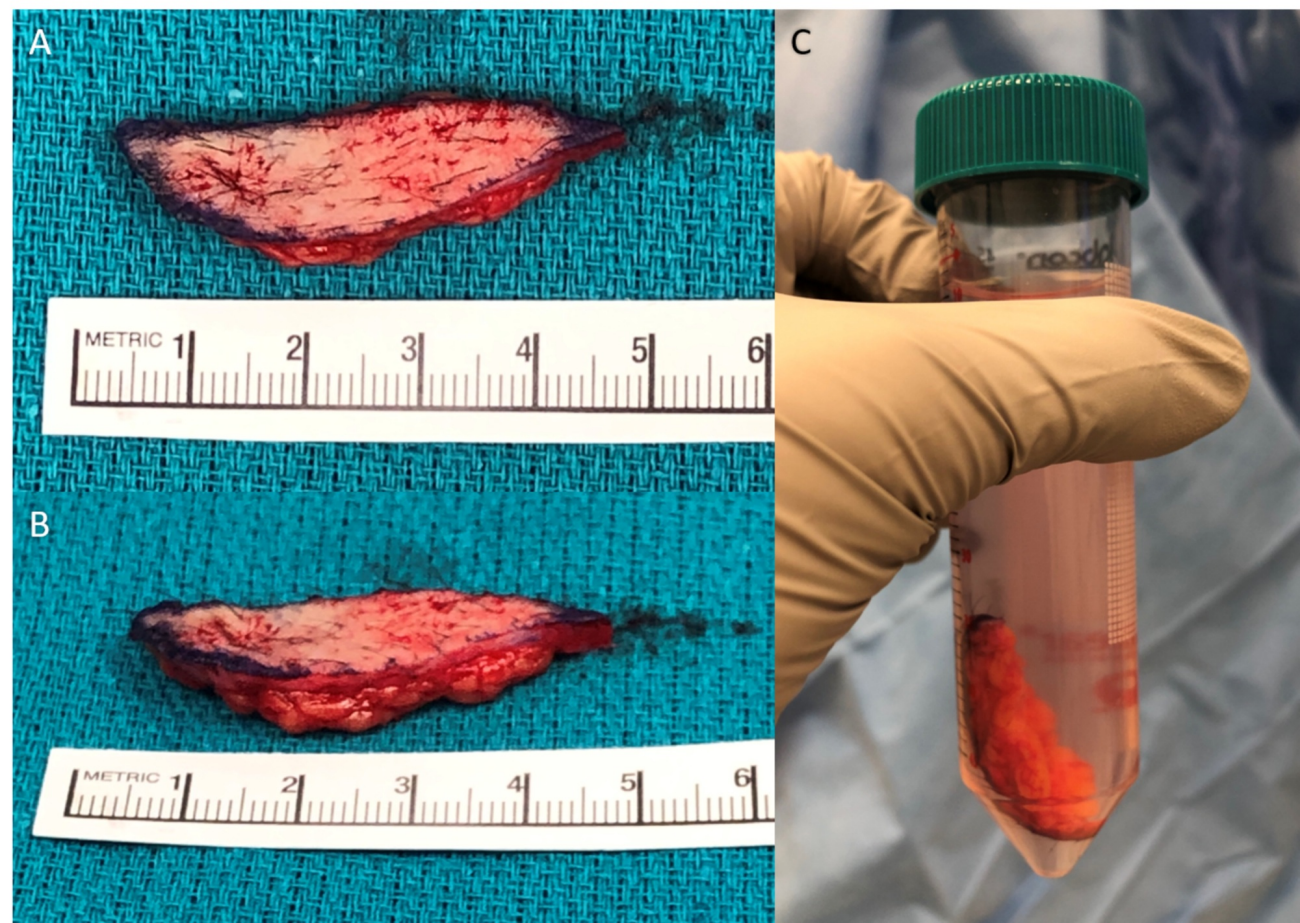
Full-thickness skin harvest Photographs showing full-thickness skin harvest taken for shipment to manufacturing facility and processing into autologous homologous skin construct. (A) Overhead image of skin harvest with ruler showing 5 x 2 cm ellipse (primary contraction resulting in slightly smaller size in picture). (B) Lateral image of skin harvest showing approximately 3-4 mm of subcutaneous fat included in harvest. (C) Image showing full-thickness skin harvest placed in sterile conical tube with approximately 40 cc of sterile normal saline for shipment.

Approximately six weeks post-injury and five weeks following application of bilayer collagen-GAG matrix, a 5 x 2 cm elliptical harvest of full-thickness skin, including a small amount of subcutaneous fat, was taken from the right groin (Figure 1) and shipped to an FDA-registered biomedical manufacturing facility, where it was processed into AHSC.

**Figure 2:**
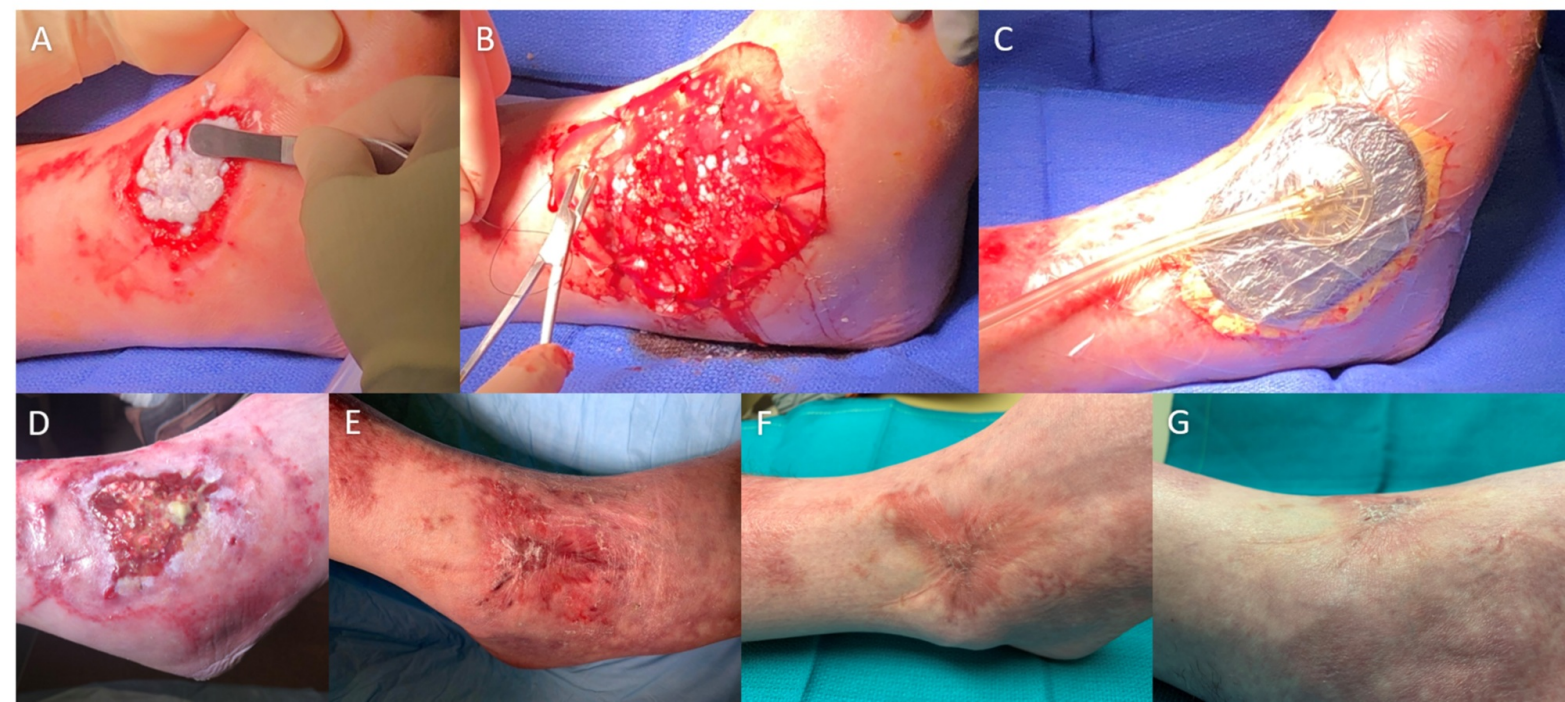
Autologous homologous skin construct (AHSC) application, dressing, and wound closure Serial photographs showing in the top row (A) application of AHSC to wound by spreading evenly with the back of forceps, (B) suturing of primary silicone dressing layer over AHSC, and (C) portable negative pressure wound therapy applied as a bolster over primary dressings. The bottom row shows the wound appearance (D) at two weeks after application of AHSC with small skin islands forming throughout center of wound, (E) complete wound closure achieved at nine weeks after AHSC application, (F) durable closure and continued skin maturation observed at 12 weeks after AHSC application, and (G) lateral angle demonstrated normal contour to surrounding skin at 12 weeks after AHSC application.

AHSC was returned six days after harvest and applied evenly across the wound seven days after harvest (Figure 2) following sharp debridement. Following application of AHSC, the wound was dressed with silicone sutured to the wound margin, covered in petrolatum gauze, and bolstered with portable negative pressure wound therapy (Figure 2). Both the harvest and application were performed in an ambulatory setting under local anesthesia. The injured ankle was immobilized in a posteriorly based thermoplastic splint, and the patient was non-weight bearing on the right, walking on crutches. Silicone was used as the primary dressing layer for 2.5 weeks after AHSC application, allowing enough time for vascular soft tissue to regenerate and cover the remaining exposed tendons. After 2.5 weeks, the primary dressing was switched to a silver non-adherent dressing (Silverlon® Wound Contact Dressing, Argentum Medical LLC, Geneva, IL) covered with an adhesive foam followed by compression with an elastic bandage (ACETM Elastic Bandage, 3M, St. Paul, MN). Nine weeks after the application of AHSC, the wound was completely closed and remained closed at most recent follow-up 16 weeks after AHSC application (Figure 2).

The percent area reduction of the wound at two weeks, six weeks, seven weeks, and nine weeks postoperative was 79%, 85%, 99%, and 100%, respectively. Out of the original 42 cm^2^ wound, there was only one small 1 cm^2^ (2.4% of original wound area) central area with mild thickening and scar formation, which did not result in functional limitations. The remainder of the wound was covered with pliable, sensate skin that had normal capillary refill. There was no restriction in tendon gliding (Video 1). The patient was taken out of the splint at eight weeks and cleared for unrestricted weight-bearing and activity at 12 weeks. Because of normal sensation and wound contour, he was able to wear normal combat boots, perform light and office-based military duties, and pursue unrestricted physical therapy. His rehabilitation trajectory is consistent with a full recovery and unrestricted military duty by nine months post-injury.

**Video 1:** Tendon gliding Video demonstrating unrestricted tendon gliding at 12 weeks following autologous homologous skin construct application.

## Discussion

This case report demonstrates for the first time the use of AHSC in treating a complex traumatic wound from an NBI in an active duty soldier. The injury occurred due to a rollover MVA in theater, resulting in a 42 cm^2^ right dorsolateral ankle wound with exposed extensor tendons. Following application of AHSC to the wound, closure was achieved at nine weeks with pliable, sensate skin and no functional restrictions or contracture. There were no donor site complications and donor site morbidity was minimal, consisting of a well-healed 5-cm linear incision in the groin.

The patient was relatively young and healthy, but did have multiple risk factors. He was an active, long-time cigarette smoker. His zone of injury was extensive, involving the foot, ankle, calf, and knee. His bony ankle fracture was treated with critical implanted fixation hardware, and extensor tendons were exposed.

Traditional treatments considered were STSG following the application of bilayer collagen-GAG matrix, tendon debridement followed by STSG, cross-leg flap, free tissue transfer, and amputation. STSG was ultimately not an option in this case because the tendons were not sufficiently covered by the collagen-GAG matrix, and it was desired to avoid tendon debridement to preserve function. The location of the wound made STSG (either after dermal matrix application or after tendon debridement) less desirable, as long-term contracture or tendon adhesions could impair ankle range of motion and require operative revision. Cross-leg flap was rejected as first-line reconstruction because of the need for prolonged bedrest, immobility, and need for coverage of the flap donor site.

Free tissue transfer was considered, but disadvantages included a prolonged operation, need for specialized equipment and hospital support, intensive care unit (ICU) postoperative monitoring, a long hospital stay, and prolonged immobilization and non-weight-bearing. Free flaps in the lower extremity, especially with an extensive zone of injury from the talus up to the knee, have a relatively high failure rate [15]. Even if the flap was successful, there would be donor site morbidities and functional issues from a bulky flap on the ankle. Debulking in order to allow the patient to wear a normal shoe or combat boot would require multiple staged operations, further prolonging disability.

Amputation would have been considered an extreme option and considered only for catastrophic treatment failure, but certainly was a possibility given the constellation of compartment syndrome, bony injury, nerve injury, and implanted hardware.

AHSC was chosen by the patient after comprehensive discussion, and in the view of the surgical team it provided a low-risk option that would not preclude the use of any traditional reconstructive options if it were to fail. Notably, both the full-thickness skin harvest and AHSC application were performed as outpatient procedures. The donor site was minimal and AHSC allowed for a 4.2-fold expansion of the harvested skin surface area. The main goal of treatment was expeditious return of function and ultimately returning the patient back to active duty, which is expected to occur by nine months post-injury after beginning unrestricted physical therapy following wound closure. Aside from the small 1 cm^2^ area of central mild scarring that caused no functional limitations, the regenerated skin is soft, supple, sensate, and with good vascularization. The patient, surgeon (O.N.J.), and medical care team were all extremely satisfied with the result achieved. After this experience using AHSC to treat a complex wound, we now have another tool in our reconstructive armamentarium. Although AHSC may not be the best choice for all wounds, we believe patient scenarios like the one in this case report may benefit from its low-risk profile and potential for return of normal function with two outpatient procedures.

The potential benefits of AHSC include minimal donor site morbidity, simple procedure, coverage of exposed structures, wound closure with functional skin that avoids contracture, contour matching to the surrounding wound bed, and fast return to pre-injury activity. These characteristics of AHSC make it a low rung on the reconstructive ladder.

## Conclusions

Military personnel deployed to a combat theater of operations sustain complex extremity wounds, both in battle and non-battle scenarios. Traditional treatments have certain limitations, due to patient factors, surgeon and facility support requirements, and post-treatment disability profile. In this case, AHSC was used to successfully treat a high-risk patient with a high-risk ankle injury. Stable wound closure, normal foot and ankle function, and hardware and tendon coverage were obtained. Additional studies are needed to characterize the effectiveness in treating combat trauma and measuring the potential contribution to readiness goals that AHSC may enable.
